# Deciphering complexity: *TULP1* variants linked to an atypical retinal dystrophy phenotype

**DOI:** 10.3389/fgene.2024.1352063

**Published:** 2024-02-21

**Authors:** Anna Esteve-Garcia, Estefania Cobos, Cristina Sau, Ariadna Padró-Miquel, Jaume Català-Mora, Pilar Barberán-Martínez, José M. Millán, Gema García-García, Cinthia Aguilera

**Affiliations:** ^1^ Department of Clinical Genetics, Bellvitge University Hospital, Institut d’Investigació Biomèdica de Bellvitge (IDIBELL), L'Hospitalet de Llobregat, Barcelona, Spain; ^2^ Department of Ophthalmology, Bellvitge University Hospital, Institut d’Investigació Biomèdica de Bellvitge (IDIBELL), L'Hospitalet de Llobregat, Barcelona, Spain; ^3^ Genetics Laboratory, Bellvitge University Hospital, Institut d’Investigació Biomèdica de Bellvitge (IDIBELL), L'Hospitalet de Llobregat, Barcelona, Spain; ^4^ Department of Ophthalmology, SJD Barcelona Children’s Hospital, Barcelona, Spain; ^5^ Molecular, Cellular, and Genomic Biomedicine Group, Valencia, Spain; ^6^ Joint Unit CIPF-IIS La Fe Molecular, Cellular and Genomic Biomedicine, Valencia, Spain; ^7^ Center for Rare Diseases (CIBERER), Madrid, Spain; ^8^ University and Polytechnic La Fe Hospital of Valencia, Valencia, Spain

**Keywords:** *TULP1*, inherited retinal dystrophy, atypical phenotype, whole-exome sequencing, minigene splice assay

## Abstract

**Introduction:** *TULP1* exemplifies the remarkable clinical and genetic heterogeneity observed in inherited retinal dystrophies. Our research describes the clinical and molecular characteristics of a patient manifesting an atypical retinal dystrophy pattern, marked by the identification of both a previously unreported and a rarely encountered *TULP1* variant.

**Methods:** Whole-exome sequencing was performed to identify potential causative variants. The pathogenicity of the identified *TULP1* variants was evaluated through *in silico* predictors and a minigene splice assay, specifically designed to assess the effect of the unreported *TULP1* variant.

**Results:** We identified two *TULP1* gene variants in a patient exhibiting unusual and symmetrical alterations in both retinas, characterized by an increase in autofluorescence along the distribution of retinal vessels. These variants included a known rare missense variant, c.1376T>C, and a novel splice site variant, c.822G>T. For the latter variant (c.822G>T), we conducted a minigene splice assay that demonstrated the incorporation of a premature stop codon. This finding suggests a likely activation of the nonsense-mediated mRNA decay mechanism, ultimately resulting in the absence of protein production from this allele. Segregation analysis confirmed that these variants were in *trans*.

**Discussion:** Our data support that individuals with biallelic *TULP1* variants may present with a unique pattern of macular degeneration and periarteriolar vascular pigmentation. This study highlights the importance of further clinical and molecular characterization of *TULP1* variants to elucidate genotype–phenotype correlations in the context of inherited retinal dystrophies.

## 1 Introduction

Inherited retinal dystrophies (IRDs) constitute a spectrum of conditions characterized by both clinical and genetic heterogeneity, affecting approximately 1 in 3,000 individuals (Hanany et al., 2020). This clinical diversity ranges from non-progressive diseases like night blindness to progressive conditions such as retinitis pigmentosa (RP) (García Bohórquez et al., 2021; Perea-Romero et al., 2021). The identification of over 300 genes conclusively linked to IRDs (RetNet, https://web.sph.uth.edu/RetNet/; accessed on 13 January 2024) also exhibits the complex nature of these conditions, marked by extensive genetic heterogeneity and variable expressivity. Notably, different variants within the same gene can lead to a wide range of clinical presentations (Chen et al., 2018; Bianco et al., 2023), and conversely, similar clinical manifestations may result from variants in different genes (García Bohórquez et al., 2021; Perea-Romero et al., 2021; Smirnov et al., 2021). Additionally, evidence supports the significant role of gene modifiers in human eye diseases (Meyer and Anderson, 2017; Li et al., 2021), introducing an added layer of complexity to the diagnosis and understanding of IRDs.

The *TULP1* gene serves as an example of the heterogeneity observed in IRDs. The link between *TULP1* and autosomal recessive IRDs was first established in 1998 (Banerjee et al., 1998; Hagstrom et al., 1998). Since then, *TULP1* biallelic variants have been extensively associated with various forms of IRDs, including non-syndromic RP, Leber congenital amaurosis, cone dystrophy, and rod–cone dystrophy (Ullah et al., 2016; Bodenbender et al., 2023). To date, the LOVD database (www.lovd.nl/gene, accessed on 13 January 2024) records a total of 117 unique *TULP1* variants. Among these, 46 are classified as variants of uncertain significance (VUS), while 106 are categorized as likely pathogenic or pathogenic.

*TULP1* encodes Tubby-like protein 1, which is a member of the TULP protein family (North et al., 1997; Nishina et al., 1998). TULP proteins have been well-documented for their critical roles in the development and function of the central nervous system (Ikeda et al., 2002). Despite displaying different expression patterns, all TULP proteins share a conserved C-terminal region of approximately 200 amino acids known as the tubby domain. TULP1, in particular, is exclusively expressed in the retina, primarily within the cytoplasm of retinal photoreceptor cells (Ikeda et al., 1999). It is assumed that TULP1 is involved in various protein–protein interactions and the intracellular protein transport, including rhodopsin and vesicle transport to and from the outer segments of photoreceptor cells (Hagstrom et al., 2001).

Bodenbender et al. (2023) reported the largest *TULP1*-related IRD cohort to date. This research established a connection between biallelic *TULP1* variants and a wide range of clinical phenotypes, emphasizing the remarkable heterogeneity in *TULP1*-related retinal dystrophy. The authors suggested that protein misfolding could be a key factor contributing to the variable clinical presentations resulting from *TULP1* gene variants. However, the intricate mechanisms underlying how TULP1 misfolding leads to specific clinical phenotypes still remain unknown (Boggon et al., 1999; Ikeda et al., 2002).

In our current research, we explore the clinical and molecular characteristics of a patient with biallelic *TULP1* variants presented with a unique pattern of macular degeneration and periarteriolar vascular pigmentation. Our primary focus lies on the identification of both a newly identified and a previously reported variant within the *TULP1* gene. This study aims to broaden the understanding of the relationship between *TULP1* and IRDs.

## 2 Materials and methods

### 2.1 Clinical evaluation

The proband underwent a comprehensive clinical assessment conducted by an experienced retinal ophthalmologist. The clinical diagnosis encompassed an array of ophthalmological evaluations, comprising visual acuity assessment, detailed fundoscopic examination, fundus photography, ultra-widefield autofluorescence (FAF) imaging, and spectral domain optical coherence tomography (SD-OCT). Electrophysiological evaluations, involving full-field flash electroretinography (ffERG) and multifocal ERG (mfERG), were also performed, following the protocols established by the International Society for Clinical Electrophysiology of Vision (ISCEV) (McCulloch et al., 2015).

The study received the ethical approval (number PR014/22) from the Ethical Committee of the University Hospital of Bellvitge and adhered to the principles outlined in the Declaration of Helsinki (World Medical Association, 2013). Written informed consent for both the genetic analysis and the publication of the paper was obtained from the participating patient.

### 2.2 Whole-exome sequencing and analysis

Whole-exome sequencing (WES) was conducted with xGen Exome Panel v2.0 (Integrated DNA Technologies, Inc., Iowa, USA). The resulting genomic library was sequenced on a NovaSeq 6000 platform (Illumina, San Diego, USA), using a 2 × 100 bp paired-end module. Subsequent bioinformatic analysis was performed using the Data Genomics Exome pipeline (version v19.1) developed by Health in Code (Valencia, Spain). For copy number variation (CNV) analysis, VarSeq software (Golden Helix, Inc., Montana, USA) was used.

WES data were filtered by a virtual gene panel comprising 295 genes (Supplementary Data S1) associated with IRDs. Only variants with a read count greater than 20 and a frequency exceeding 30% were considered. Furthermore, variants were filtered based on a minor allele frequency of 1/500 in gnomAD v2.1.1 (Karczewski et al., 2020).

Variants showing a predicted deleterious effect on the encoded protein were retained for further analyses, with priority given to nonsense, frameshift, splice site, and missense variants. The impact of missense variants was evaluated through the REVEL *in silico* tool (Ioannidis et al., 2016). For assessing splice site variants, the following bioinformatics tools were used: SpliceAI (Jaganathan et al., 2019), MaxEntScan, and SPiP. The latter predictors were used through the MobiDetails platform (https://mobidetails.iurc.montp.inserm.fr/MD/) (Baux et al., 2021). The NMDEscPredictor was used to predict whether the transcript might be subject to degradation by nonsense-mediated decay (NMD) or not (https://nmdprediction.shinyapps.io/nmdescpredictor/) (Coban-Akdemir et al., 2018).

To validate the identified variants and investigate their segregation, Sanger sequencing was used. This also aimed to ascertain whether the variants coexisted in the same allele (*cis* configuration) or in separate alleles (*trans* configuration).

### 2.3 Minigene splice assay

To confirm the pathogenicity of the *TULP1*(NM_003322.6):c.822G>T variant, which has the potential to impact splicing, a minigene splice assay was conducted. The assay was based on a previously described protocol (Rodriguez-Muñoz et al., 2022).

The exon containing the variant (exon 8) and approximately 250 bp of the adjacent 5′ and 3′ intronic regions were amplified from the patient’s DNA using the Phusion High-Fidelity Polymerase enzyme (Thermo Fisher Scientific, Waltham, MA, USA). The primers used for amplification also incorporated the restriction sites for the XhoI and NheI enzymes (Supplementary Table S1). These enzymes were subsequently used to digest both the pSPL3 vector (kindly provided by Dr. I. Botillo and Dr. S. Tufery-Giraud) and the purified amplicon. Following the purification of the digested products, the amplicon was inserted into the plasmid using T4 DNA ligase (Thermo Fisher Scientific). NEB Stable competent *Escherichia coli* cells were transformed with vector construction by electroporation. Wild-type and mutant constructs were confirmed by Sanger sequencing. Then, two replicates of the transfection were carried out as follows: 500 ng of wild-type and mutant constructs were separately transfected in HEK293 cells using the Lipofectamine™ 3000 reagent (Thermo Fisher Scientific). After 24 h, the cells were collected, and total RNA was isolated using the RNeasy Mini kit (QIAGEN, Hilden, Germany). RT-PCR was performed using the PrimeScript RT Reagent Kit (TaKaRa, Kusatsu, Japan). The cDNA was amplified with the FIREPol DNA Polymerase enzyme (Solis BioDyne, Tartu, Estonia) using pSPL3-specific primers. The products were separated on a 2% agarose gel, and bands were purified with the QIAquick Gel Extraction Kit (QIAGEN, Hilden, Germany). The results were analyzed by Sanger sequencing.

## 3 Results

### 3.1 Clinical description

**FIGURE 1:**
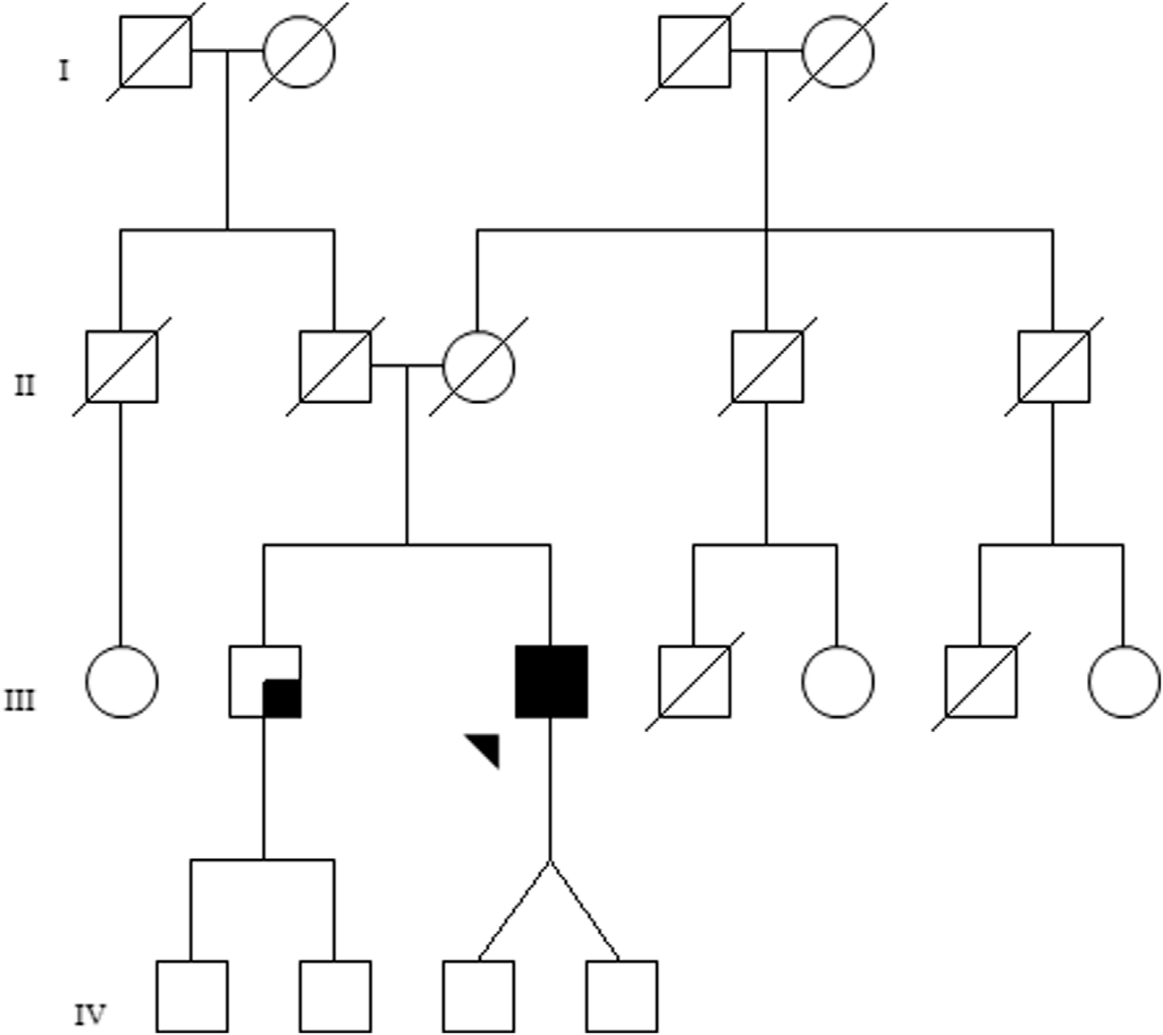
Family pedigree of the investigated patient. The proband (III-3) is marked by an arrow, and the black shaded square represents bilateral ophthalmological disease. The brother of the patient (III-2) presents with visual loss in the left eye, not suggestive of retinal dystrophy, which is represented by a small shaded square.

The family pedigree is shown in Figure 1. The proband, identified as III.3, is a 51-year-old Caucasian male who presented at our joint Ophthalmology–Genetics Clinic with a 10-year history of bilateral progressive vision loss and photophobia. He mentioned experiencing nyctalopia from the age of 20, which he attributed to his preexisting myopia and astigmatism. The patient’s brother, a 59-year-old man, referred to be experiencing visual impairment in his left eye. No other relevant ophthalmological family history was noted. The proband has two asymptomatic children.

Upon the initial ophthalmic evaluation of the proband, Snellen visual acuity measurements were 20/800 in the right eye (OD) and 20/1600 in the left eye (OS). Anterior segment biomicroscopy revealed the presence of bilateral nuclear cataracts. Fundus examination unveiled a distinctive pattern of retinal pathology with an extensive atrophy of the retinal pigment epithelium, notably between the arcades in the posterior pole accompanied by a mild preservation of the perifoveal retina, characteristic of a bull’s eye maculopathy. The atrophic changes extended into the nasal region, progressing toward the equatorial retina, where some intraretinal spicule-shaped pigmentary deposits were discernible.

**FIGURE 2:**
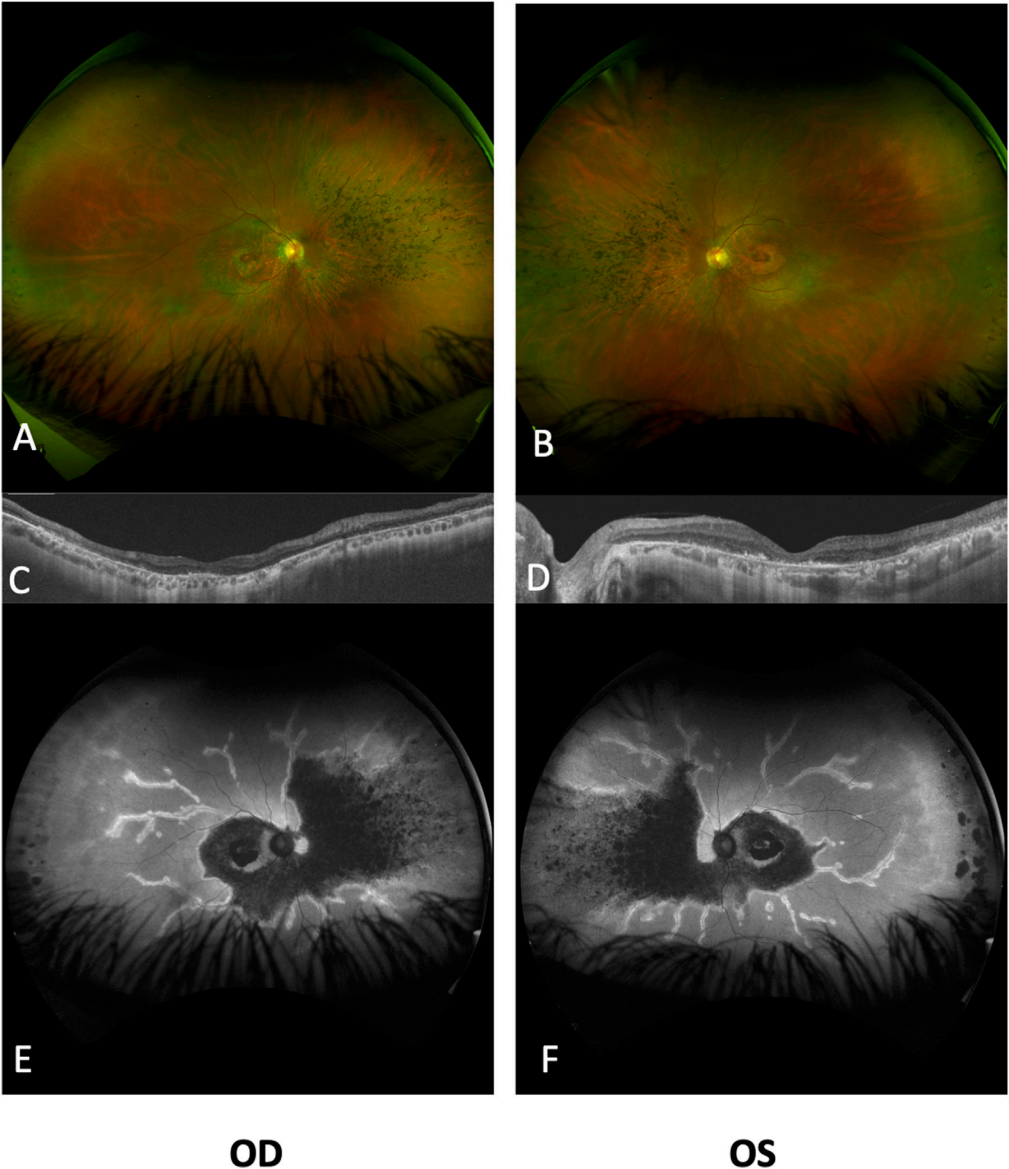
Ultra-widefield fundus imaging and OCT analysis of the retinal changes in both eyes. Ultra-widefield fundus photographs of the right **(A)** and left **(B)** eyes, revealing significant retinal atrophy surrounding the optic disc, affecting both superior and inferior vascular arcades. Additionally, bone spicules are evident in the nasal quadrant, accompanied by narrowed vessels. Notably, both eyes display a perifoveal ring of atrophy within the macular area. Subretinal optical coherence tomography (ssOCT) scans of the right **(C)** and left **(D)** eyes reveal a pronounced loss of the ellipsoid layer in the outer segments, with a mild degree of preservation observed in the subfoveal photoreceptors. The inner retinal layers exhibit relatively better preservation. Ultra-widefield autofluorescence (FAF) images for the right **(E)** and left **(F)** eyes highlight marked reductions in FAF intensity within the posterior pole and nasal retinal regions, including the peripapillary area, with minimal FAF preservation evident in the foveal region. The perivascular retina exhibits increased FAF extending from the posterior pole to the mid-peripheral regions. OD, right eye; OS, left eye.

**FIGURE 3:**
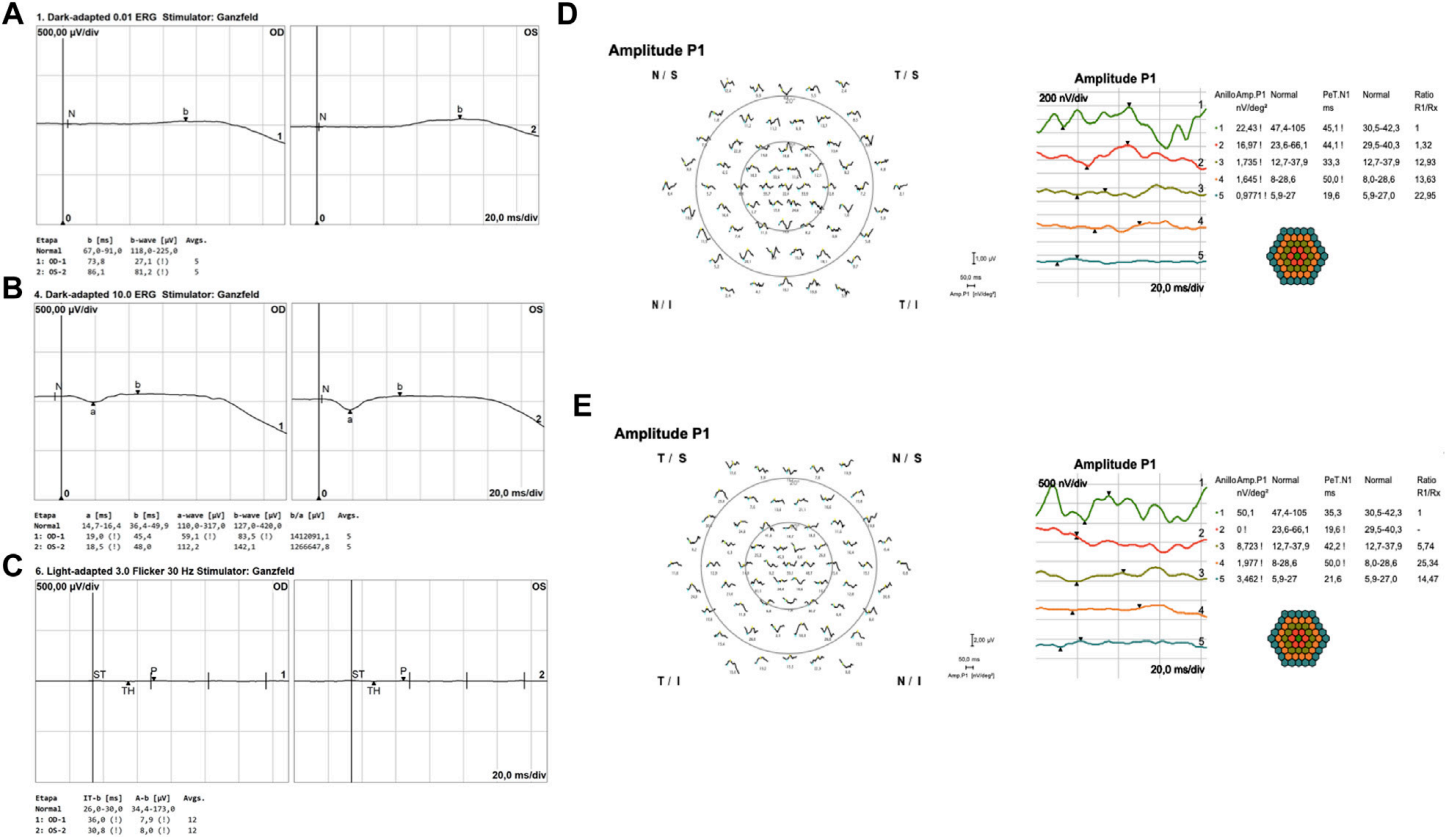
Full-field flash electroretinography (ffERG) and multifocal ERG (mfERG) results. In panels **(A, B)**, dark-adapted ERG, adhering to ISCEV standards, was recorded using Dawson, Trick, and Litzkow (DTL) electrodes. After the DA 0.01 cd-s/m^2^ stimulus, both eyes exhibited a flat response **(A)**. DA 10.0 cd-s/m^2^ response showed an increase in implicit time and a reduction in amplitude for both **(A, B)** waves, with the differences being more pronounced in the right eye. Panel **(C)** illustrates the light-adapted protocol using DTL electrodes, wherein the 30-Hz flicker ERG demonstrated a flat response. Panels **(D, E)** showcase multifocal ERG results for the right **(D)** and left **(E)** eyes, recorded with jet corneal electrodes. These results indicate a decrease in P1 amplitudes in both central and peripheral rings, with minimal foveal sparing observed in the first ring.

SD-OCT imaging confirmed foveal thinning, along with evident distortion and the loss of the outer nuclear layer, external limiting membrane, ellipsoid zone, and retinal pigment epithelium bilaterally. FAF imaging revealed a well-defined hypofluorescent area in the posterior pole and nasal retina, encircled by a hyperautofluorescent border. Additionally, a prominent increase in autofluorescence around the retinal arteries was observed in the equatorial retina, extending into the mid-peripheral region (Figure 2). The ffERG results revealed a diminished response in both scotopic and photopic protocols. MfERG displayed a notable absence of responses in the four peripheral rings, with residual responses in the central ring showing reduced amplitudes (Figure 3).

Comprehensive evaluation of the ophthalmic assessments and clinical symptoms led to the clinical diagnosis of cone–rod dystrophy in the patient.

An ophthalmic examination of the proband’s brother, a 59-year-old man, revealed scarring with loss of macular photoreceptors in the left eye, without affecting the right eye, which is not suggestive of retinal dystrophy.

### 3.2 Identification of biallelic variants in the *TULP1* gene

WES analysis identified a total of 36 variants after filtering the exome data for the 295 candidate IRD genes and applying a population allele frequency threshold of 1/500. Among these variants, only four were predicted to have potential deleterious effects on the corresponding proteins according to the REVEL score (Supplementary Table S2). Finally, two variants, *GUCY2D* (NM_000180.4):c.1664A>G and *NPHP4*(NM_015102.5):c.3630T>G, were excluded from further considerations. The former showed non-segregation with the disease within the family, while the latter was identified in a gene that follows an autosomal recessive inheritance pattern, lacking a second detected variant.

Two heterozygous variants within the *TULP1* gene emerged as the most promising candidates: an unreported variant, NM_003322.6:c.822G>T, and a previously described variant, NM_003322.6:c.1376T>C (Supplementary Table S2). The variant c.822G>T is a novel alteration located in exon 8/15, resulting on the substitution of a highly conserved lysine with asparagine at position 274 of the protein NP_003313.3:p.(Lys274Asn), located within the disordered domain of TULP1 (Supplementary Table S2). Importantly, this substitution is located on the final nucleotide of exon 8 and is predicted to impact the splicing process. *In silico* splicing predictors suggested that the variant was likely to affect splicing (SpliceAI score 0.92, SPiP score 0.976, and MaxEntScan score −10.19), potentially leading to the loss of a donor splicing site and causing an exon skipping or the utilization of a cryptic splice site located eight nucleotides upstream (PP3-supporting). The variant was absent from gnomAD (PM2-supporting) (Supplementary Table S3). In line with the ACMG/AMP guidelines (Richards et al., 2015), the variant was initially classified as a variant of uncertain significance.

The second variant identified, c.1376T>C, is located in exon 14/15 within the *TULP1* gene, resulting in the amino acid substitution of a strongly conserved isoleucine with threonine at position 459 of the protein NP_003313.3:p. (Ile459Thr) (Supplementary Figure S1). This variant exhibits a low frequency in gnomAD v2.1.1 (allele frequency 0.000077 in the European non-Finnish population) (PM2-supporting). The *in silico* predictor REVEL suggests a deleterious impact on the protein, with a score of 0.865 (PP3-moderate). Notably, this variant is situated within the tubby domain, a known hotspot for *TULP1* variants (PM1-moderate). Furthermore, an alternative variant (chr6:35467877A>T, p.(Ile459Thr)) has been reported as pathogenic in the UniProt database (Bateman et al., 2023) (PM5-moderate). This variant has also been documented in the ClinVar database (Landrum et al., 2018) (VCV000194380.14), where it is classified as a variant of uncertain significance and likely benign (Supplementary Table S3). Additionally, the variant has been previously reported in several scientific articles (Wang et al., 2014; Ullah et al., 2016; Chen et al., 2018). In accordance with the ACMG/AMP guidelines, the variant was classified as likely pathogenic.

The segregation study conducted in the patient’s brother demonstrated that the variants were located in different alleles (*trans* configuration) (Supplementary Figure S2). Although this finding supported the use of the PM3-moderate criterion for classifying the c.822G>T variant, it still remained classified as a variant of uncertain significance.

### 3.3 Minigene splice assay results

All the *in silico* predictors mentioned above predicted an aberrant splicing of the *TULP1*(NM_003322.6):c.822G>T variant. To validate the variant’s functional impact, a minigene splice assay was conducted.

**FIGURE 4:**
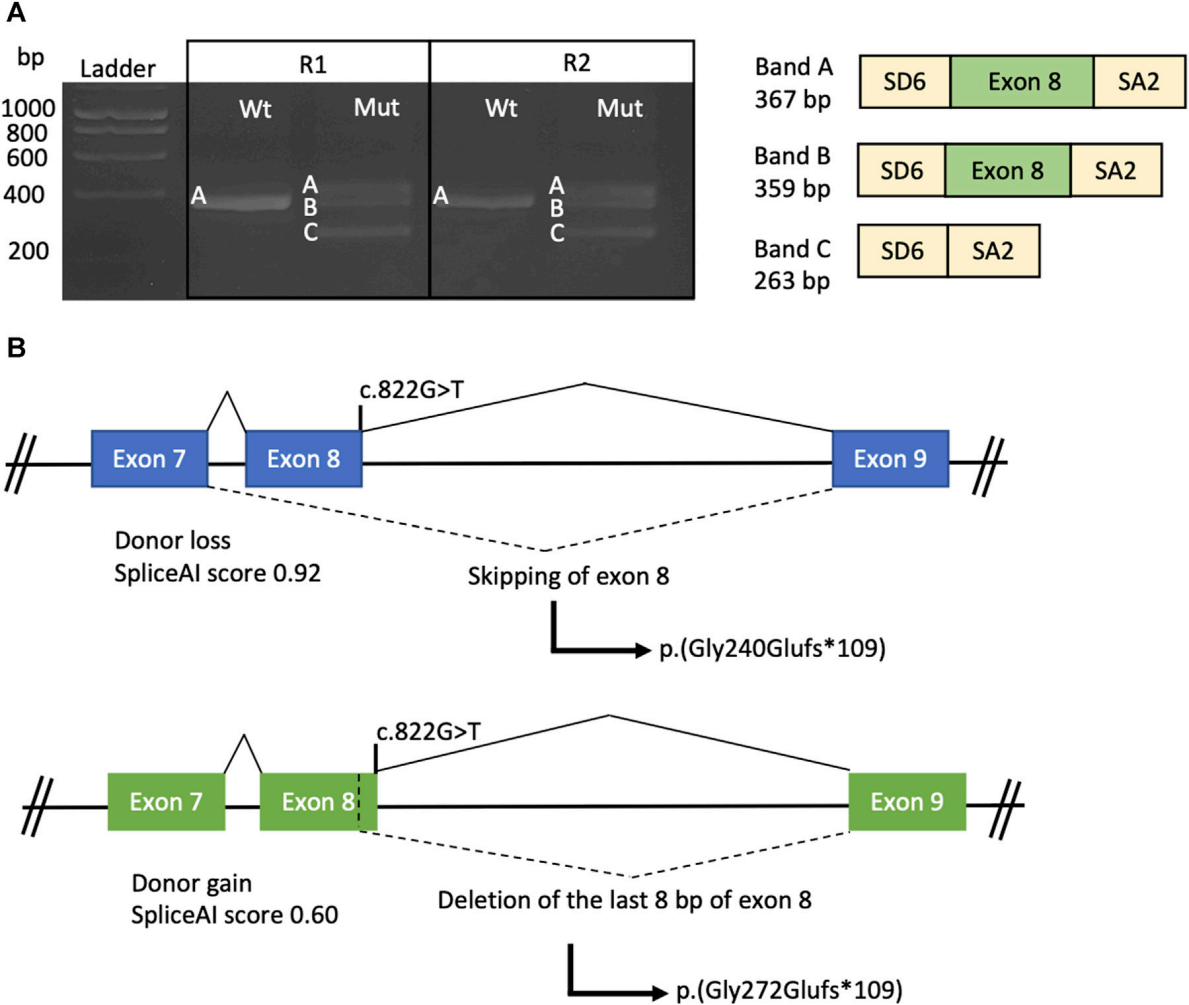
Minigene splice assay conducted on the *TULP1*:c.822G>T variant. **(A)** Agarose gel electrophoresis of the products obtained by RT-PCR from both the wild-type (Wt) and mutated (Mut) constructs, with experiments performed in duplicate (R1 and R2). On the right side, a representation of the amplified products is provided, with yellow boxes representing the SD6 and SA2 exons from pSPL3 and the green box representing exon 8 of the *TULP1* gene. **(B)** Schematic representation of the *TULP1* gene exons 7, 8, and 9 illustrates the predicted impact of the c.822G>T variant on splicing. The upper scheme depicts the molecular effects of exon 8 skipping, while the lower scheme illustrates the loss of the last 8 bp of exon 8. Exons are depicted as boxes, and introns are represented as lines.

In the wild-type minigene, a fragment corresponding to the wild-type mRNA was observed (Figure 4A, band A—Wt). However, in the mutant minigene, three transcripts were observed: the wild-type transcript (Figure 4A, band A—Mut) and two aberrant transcripts. One aberrant transcript results from the exclusion of exon 8 attributed to the loss of the native donor splice site (Figure 4A, band C—Mut) (SpliceAI: 0.92). Conversely, the alternative band encompasses exon 8, except for the final 8 bp, stemming from the activation of a cryptic donor site (Figure 4A, band B—Mut) (SpliceAI: 0.60).

Regarding the protein-level effect, both aberrant transcripts would generate a change in the reading frame, resulting in a premature stop codon and a truncated protein (Figure 4B). Therefore, mRNA degradation of both transcripts could occur through NMD, according to the NMDEscPredictor.

Based on these findings, the NM_003322.6:c.822G>T variant was reclassified as pathogenic as it met the criteria for PVS1 in accordance with the ClinGen SVI splicing group’s recommendations (Walker et al., 2023).

## 4 Discussion

TULP1 is a crucial retinal protein for intracellular protein transport within photoreceptor cells. It has been associated with a wide range of retinal disorders including RP and early-onset RP, Leber congenital amaurosis, cone dystrophy, and rod–cone dystrophy (Banerjee et al., 1998; Hagstrom et al., 1998; Lobo et al., 2016; Woodard et al., 2021; den Hollander et al., 2007; Jacobson et al., 2014). Despite the diversity of associated conditions, consistent differences in clinical presentation attributed to specific *TULP1* variants, or their impacts on the protein have not been reported (North et al., 1997; Lobo et al., 2016; Remez et al., 2020; Jia et al., 2022). Notably, it has been suggested that patients carrying homozygous or compound heterozygous *TULP1* pathogenic variants, particularly those affecting the tubby domain and/or resulting in a loss-of-function (LOF) effect, typically exhibit a severe, early-onset form of retinal dystrophy (den Hollander et al., 2007; Jacobson et al., 2014; Jia et al., 2022; Mataftsi et al., 2007).

Here, we describe a patient presenting a novel *TULP1* gene variant (c.822G>T), which has been predicted to disrupt the splicing process. The variant is located within the disordered domain, for which structural information is currently lacking (Boggon et al., 1999; Lobo et al., 2016). In a hypothetical scenario where the protein is synthesized, it is possible that the tubby domain might be absent. Our minigene splice assays demonstrated that the c.822G>T variant induces exon 8 skipping, resulting in the formation of a premature stop codon p.(Gly240Glufs*109). Alternatively, there is a possibility that, instead of exon skipping, the last eight base pairs of exon 8 are omitted, thereby leading to a premature stop codon p.(Gly272Glufs*109). In both scenarios, the introduction of a premature stop codon is likely to activate the NMD mechanism, ultimately resulting in the absence of protein production from this allele (Figure 4B). Similarly, Bodenbender et al. (2023) performed minigene assays for the c.1495+1G>A and c.1496–6C>A *TULP1* variants. In both cases, the variants were predicted to induce frameshift variants and premature termination codons, leading to the probable degradation of the mutant transcript via the NMD mechanism. The first variant achieved this by inducing exon 14 skipping and the insertion of 17 amino acids p.(Ala442Profs*18), while the second variant activated a cryptic acceptor site p.(Pro499Leufs*143). Further functional studies would be required in retinal cells to establish the pathogenic mechanism, considering that splicing is specific to the tissue.

On the other hand, the c.1376T>C, p.(Ile459Thr) variant induces a substitution at amino acid position 459, replacing an isoleucine with a threonine. This change entails a shift from a hydrophobic side chain to a polar one, which could potentially affect protein stability or induce misfolding (Lobo et al., 2016). The variant p.(Ile459Thr) is located in the tubby domain of TULP1, which has previously been shown to exhibit DNA-binding activity and function as a transcription factor (Carroll et al., 2004). Missense variants situated in the tubby domain are expected to accumulate at the endoplasmic reticulum, potentially activating the unfolded protein response, which can promote apoptosis and photoreceptor cell death (Jia et al., 2022). Interestingly, the variant c.1376T>C has been previously reported in *trans* with the c.1112+2T>C variant in a patient with non-syndromic RP (Wang et al., 2014). Although both our patient and the patient reported by Wang et al. (2014) present the same missense variant (c.1376T>C) affecting the tubby domain and another variant that causes LOF, their phenotypes are markedly distinct, illustrating the significant variability in patients with *TULP1* variants.

**FIGURE 5:**
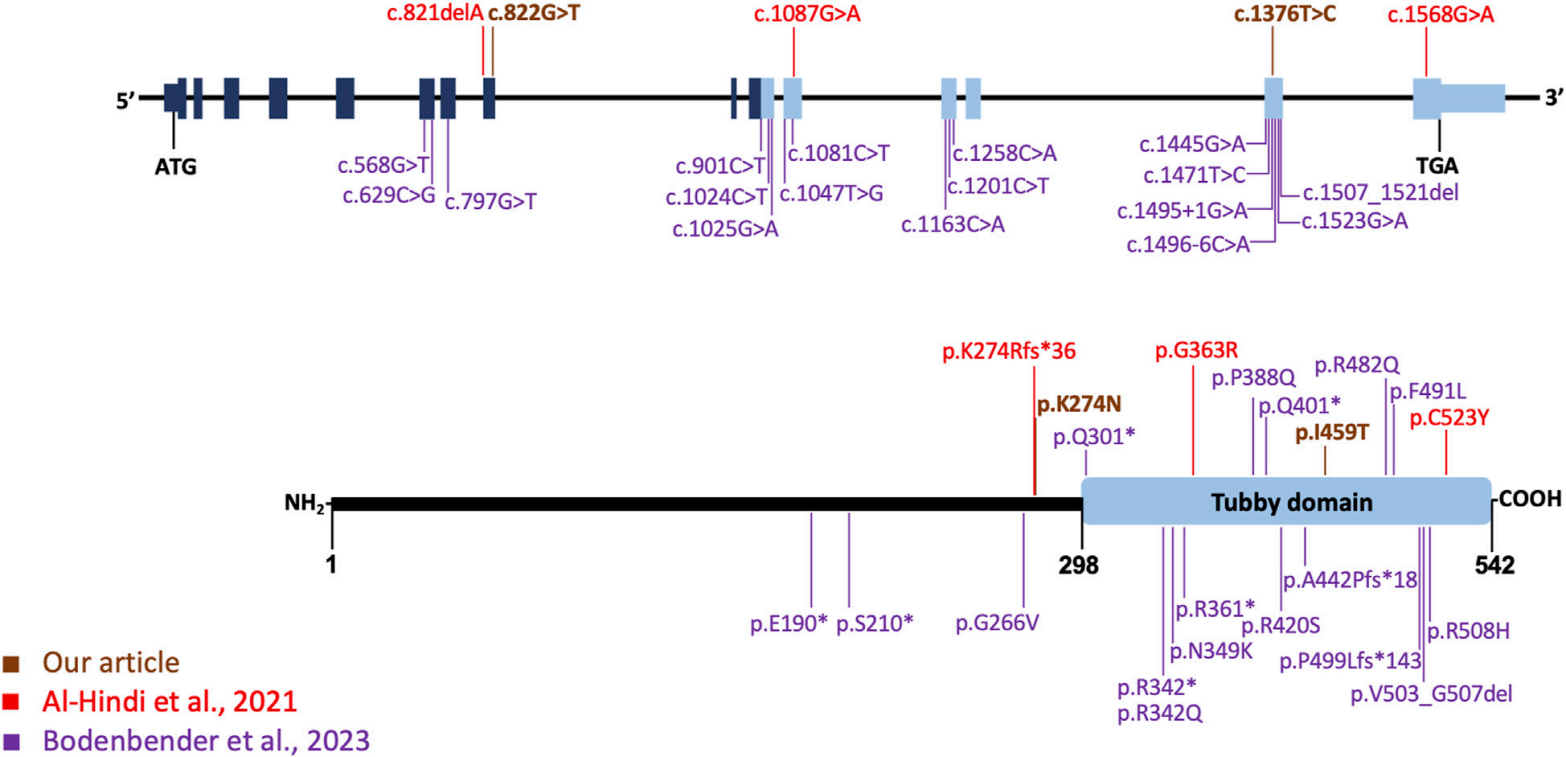
*TULP1* gene, its protein structure, and the distribution of pathogenic variants. In the upper section, the gene organization is depicted, along with the *TULP1* variants identified in this study (highlighted in dark orange) and those reported by Al-Hindi et al. (2021) (in red) and the 17 variants by Bodenbender et al. (2023), represented in purple. The lower section provides a schematic representation of the tubby-like protein 1 domain structure, with variants from Al-Hindi et al. (2021) shown in red, those from Bodenbender et al. (2023) in purple, and the variants from our study displayed in dark orange. The tubby domain is indicated by a light blue box at the C-terminal.

Our case exhibits bull’s eye maculopathy characterized by perivascular pigmentation and increased autofluorescence around retinal vessels (Figure 2). This phenotype corresponds to observations detailed by Al-Hindi et al. (2022), who reported a similar distinctive phenotype in two patients with cone dysfunction and a perivascular pattern of retinal degeneration. Case 1 in Al-Hindi et al. (2022) displays a clinical and FAF appearance remarkably similar to our patient, both in progression and test outcomes. However, notable differences exist, such as a greater degree of macular atrophy in our patient, potentially linked to the older age of our subject (51 years vs. 19 years). Remarkably, case 1 is a homozygous carrier of the p. (Gly363Arg) missense variant impacting the tubby domain (Figure 5). The second case in Al-Hindi et al. (2022) shares similarities with our case in terms of the bull’s eye maculopathy. The FAF also suggests periarteriolar hyperautofluorescence. Interestingly, case 2 shares a genotype composition similar to our patient, carrying a missense variant (p.(Cys523Tyr)) affecting the tubby domain, alongside a LOF variant (p.(Lys274ArgfsTer36)). In contrast to cases described by Al-Hindi et al. (2022), our patient lacks pigment clumps surrounding vessels and shows no signs of perivascular choroidal atrophy. However, the distinct pattern of periarterial degeneration observed in both our case and Al-Hindi et al. (2022) cases has not been documented in other studies.

Bodenbender et al. (2023) reported a total of 17 different *TULP1* pathogenic variants, categorized as missense, splice site, and nonsense variants, and one in-frame deletion. All documented variants, except the missense variant p.(Gly266Val), either affected the tubby domain or led to LOF (Figure 5). The p.(Gly266Val) variant, identified in *trans* with a LOF *TULP1* variant, was associated with cone dystrophy in a male patient (P15 in their series) diagnosed at age 40. This suggests that missense variants like p.(Gly266Val), not affecting the tubby domain, may result in a relatively mild reduction of protein activity and a later-onset phenotype. When comparing our case with those in Bodenbender et al. (2023) series, participant P17 stands out as the most analogous, exhibiting bull’s eye maculopathy. Although P17’s retinography only captures the central 50°, there is no apparent perivascular hyperautofluorescence pattern. Similarly, on OCT, mirroring our case, outer segments are visible, although with foveal discontinuity.

In this study, we present a single case of a patient with compound heterozygous *TULP1* variants. The prevalence of biallelic pathogenic *TULP1* variants is remarkably low, accounting for less than 1% of reported non-syndromic IRD cases, as documented in GeneReviews (Fahim et al., 2023; Kumaran et al., 2023). Within our extensive cohort of over 1,000 pediatric and adult patients diagnosed with IRDs, we have identified an additional case, distinct from the one presented here, with confirmed *TULP1* pathogenic variants (internal data). Our findings align with the existing literature, which documents only 78 patients with *TULP1*-associated IRDs from 44 families (Hagstrom et al., 1998; Ullah et al., 2016; Bodenbender et al., 2023; Woodard et al., 2021; den Hollander et al., 2007). This collective evidence underscores the limited occurrence of such cases, reinforcing the distinctive nature of our reported case.

Additional factors have been suggested to contribute to the variable clinical presentation observed in *TULP1*-related IRDs, including protein misfolding (Bodenbender et al., 2023), environmental factors or the influence of genetic modifiers (Meyer and Anderson, 2017). *MAP1A* has been suggested as a modifier gene for *TULP1* in a mouse model (Maddox et al., 2012; Youn et al., 2022), offering protective effects and elucidating variations in disease manifestation. However, the analysis of the *MAP1A* gene in our patient revealed five homozygous variants (Supplementary Table S4) with a high population frequency, predicted to have no impact on the protein. Consequently, these variants may not significantly influence the patient’s phenotype. Nevertheless, exploring the *MAP1A* gene on all patients with biallelic variants in *TULP1* would be of great interest to determine if there are specific variants associated with a protective effect or those linked to a more severe phenotype.

To gain a deeper insight into the pathogenesis and variations in the phenotype, further investigations should be conducted using animal models of the retina. Additionally, these studies can contribute to the development of gene therapies, similar to those that have been successfully developed for other IRDs (Russell et al., 2017). Efforts have been made for *TULP1*, although a supplementation therapy trial targeting photoreceptors in Tulp1^−/−^ mouse retinas demonstrated limited effectiveness (Palfi et al., 2020). This study is still crucial to increase our understanding of the phenotypic traits of the disease. This knowledge forms the basis for early diagnosis and future therapies.

In conclusion, we described a patient with biallelic *TULP1* variants, displaying an atypical perivascular pattern of retinal degeneration. Furthermore, we illustrated that the variant c.822G>T induces aberrant splicing, resulting in either exon 8 skipping or the loss of the last 8 bp of exon 8, both culminating in LOF. The molecular characterization of the c.822G>T variant allowed its reclassification, establishing its pathogenic effect. This highlights the importance of conducting functional studies on variants situated outside canonical splice sites that still show potential to impact splicing.

## Data Availability

The datasets presented in this study can be found in online repositories. The names of the repository/repositories and accession number(s) can be found in Supplementary Material.
