# Supplementary material for: Deciphering complexity: TULP1 variants linked to an atypical retinal dystrophy phenotype
Source: Front Genet. 2024 Feb 21;15:1352063. doi: 10.3389/fgene.2024.1352063 (PMC10915255; doi:10.3389/fgene.2024.1352063)
Supplement: Supplementary file 1 [file DataSheet1.pdf]

Supplementary Material

1 Supplementary Data

List of the 295 genes currently associated with inherited retinal dystrophies sourced from RetNet (https://web.sph.uth.edu/RetNet/; accessed on January 2024):

ABCA4, ABCC6, ABHD12, ACBD5, ACO2, ADAM9, ADAMTS18, ADGRA3, ADGRV1, ADIPOR1, AFG3L2, AGBL5, AHI1, AHR, AIPL1, ALMS1, ARHGEF18, ARL2BP, ARL3, ARL6, ARMS2, ARSG, ASRGL1, ATF6, ATOH7, ATXN7, BBIP1, BBS1, BBS10, BBS12, BBS2, BBS4, BBS5, BBS7, BBS9, BCM, BEST1, C1QTNF5, C2, C3, CA4, CABP4, CACNA1F, CACNA2D4, CAPN5, CC2D2A, CCT2, CDH15, CDH23, CDHR1, CEP164, CEP19, CEP250, CEP290, CEP78, CERKL, CFAP410, CFAP418, CFB, CFH, CHM, CIB2, CISD2, CLCC1, CLN3, CLRN1, CLUAP1, CNGA1, CNGA3, CNGB1, CNGB3, CNNM4, COG6, COL11A1, COL2A1, COL9A1, CRB1, CRX, CSPP1, CTNNA1, CWC27, CYP4V2, DHDDS, DHX38, DMD, DRAM2, DTHD1, DYNC2H1, DYNC2I2, EFEMP1, ELOVL1, ELOVL4, EMC1, ENSA, ERCC6, ESPN, EXOSC2, EYS, FBLN5, FLVCR1, FSCN2, FZD4, GDF6, GNAT1, GNAT2, GNB3, GNPTG, GPR179, GRK1, GRM6, GUCA1A, GUCA1B, GUCY2D, HARS1, HGSNAT, HK1, HMCN1, HMX1, HTRA1, IDH3B, IFT140, IFT172, IFT27, IFT81, IMPDH1, IMPG1, IMPG2, INVS, IQCB1, ITM2B, JAG1, KCNJ13, KCNV2, KIAA1549, KIF11, KIF3B, KIZ, KLHL7, LAMA1, LCA5, LRAT, LRIT3, LRP5, LZTFL1, MAK, MAPKAPK3, MERTK, MFN2, MFRP, MFSD8, MIEF1, MIR204, MKKS, MKS1, MMP19, MT-ATP6, MT-ND4, MTRFR, MT-TH, MT-TL1, MT-TP, MT-TS2, MVK, MYO7A, NBAS, NDP, NEK2, NEUROD1, NMNAT1, NPHP1, NPHP3, NPHP4, NR2E3, NR2F1, NRL, NYX, OAT, OFD1, OPA1, OPA3, OPN1LW, OPN1MW, OPN1SW, OR2W3, OTX2, PANK2, PAX2, PCARE, PCDH15, PCYT1A, PDE6A, PDE6B, PDE6C, PDE6G, PDE6H, PDZD7, PEX1, PEX2, PEX7, PGK1, PHYH, PITPNM3, PLA2G5, PLK4, PMPCA, PNPLA6, POC1B, POC5, POMGNT1, PPT1, PRCD, PRDM13, PROM1, PROS1, PRPF3, PRPF31, PRPF4, PRPF6, PRPF8, PRPH2, PRPS1, RAB28, RAX2, RB1, RBP3, RBP4, RCBTB1, RD3, RDH11, RDH12, RDH5, REEP6, RGR, RGS9, RGS9BP, RHO, RIMS1, RIMS2, RLBP1, ROM1, RP1, RP1L1, RP2, RP9, RPE65, RPGR, RPGRIP1, RPGRIP1L, RS1, RTN4IP1, SAG, SAMD11, SDCCAG8, SEMA4A, SLC24A1, SLC25A46, SLC38A8, SLC4A7, SLC7A14, SNRNP200, SPATA7, SPP2, TEAD1, TIMM8A, TIMP3, TLR3, TLR4, TMEM126A, TMEM216, TMEM237, TOPORS, TREX1, TRIM32, TRNT1, TRPM1, TSPAN12, TTC8, TTLL10, TTPA, TUB, TUBGCP4, TUBGCP6, TULP1, UNC119, USH1C, USH1G, USH2A, VCAN, WDPCP, WDR19, WFS1, WHRN, ZNF408, ZNF423, ZNF513.

2 Supplementary Figures and Tables

2.1 Supplementary Tables

Supplementary Table S1. Primers used to amplify the *TULP1*:c.822G>T variant. The tail added to the primers is indicated in lowercase letters and the restriction sites of the enzymes are indicated in bold in both the direct primer XhoI and in the reverse NheI.

Primer	Sequence (5’ to 3’)
TULP1_8D_minigen	aagaat**CTCGAG**GCATACAAGACAGTGCTAGG
TULP1_8R_minigen	aagaat**GCTAGC**AGAGGGGAGGCCTCAGAG

Supplementary Table S2. Variants obtained after filtering WES data.

Gene	Genomic coordinates (hg19)	Nucleotide change	Amino acid change	Variant type	Zigosity	ACMG classification
GUCY2D	chr17:7911346A>G	NM_000180.4:c.1664A>G	NP_000171.1:p.(Tyr555Cys)	Missense	Het	Variant of uncertain significance
NPHP4	chr1:5926447A>C	NM_015102.5:c.3630T>G	NP_055917.1:p.(Phe1210Leu)	Missense	Het	Variant of uncertain significance
TULP1	chr6:35476986C>A	NM_003322.6:c.822G>T	NP_003313.3:p.(Lys274Asn)	Missense	Het	Variant of uncertain significance
TULP1	chr6:35467877A>G	NM_003322.6:c.1376T>C	NP_003313.3:p.(Ile459Thr)	Missense	Het	Likely pathogenic

Supplementary Table S3. *TULP1* variants identified in our patient.

Nucleotide change (NM_003322.6)	Amino acid change (NP_003313.3)	Exon location	Variant type	dbSNP	gnomAD MAF	REVEL score	SpliceAI score	Conservation score PhyloP100	Published
c.822G>T	p.(Lys274Asn)	8/15	missense	-	-	-	0.92	4.522	No
c.1376T>C	p.(Ile459Thr)	14/15	missense	rs121909075	0.000077	0.865	-	7.491	Yes (4;22;23)

gnomAD, Genome Aggregation Database; MAF, minor allele frequency; REVEL, Rare Exome Variant Ensemble Learner

Supplementary Table S4. Variants identified in the modifier gene *MAP1A*.

Nucleotide change (NM_002373.5)	Amino acid change (NP_002364.5)	Exon location	Variant type	Zigosity	dbSNP	gnomAD MAF	REVEL score	SpliceAI score
c.3733G>A	p.(Asp1245Asn)	4/6	missense	Hom	rs12912505	0.1529	0.108	-
c.4408G>A	p.(Ala1470Thr)	4/6	missense	Hom	rs62020612	0.16313	0.039	-
c.2328C>T	p.(Pro776Pro)	4/6	synonymous	Hom	rs3862138	0.16145	-	0
c.3246G>A	p.(Gly1082Gly)	4/6	synonymous	Hom	rs1060939	0.35786	-	0
c.3735T>C	p.(Asp1245Asp)	4/6	synonymous	Hom	rs480108	0.35784	-	0

2.2 Supplementary Figures

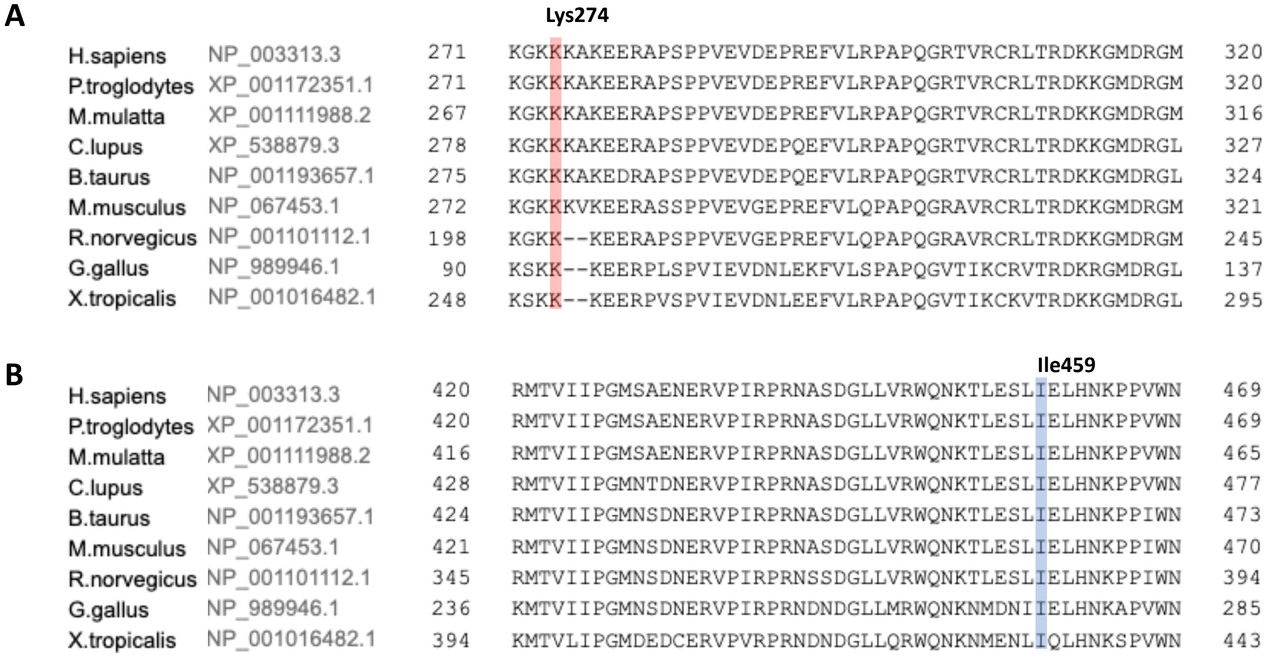

Supplementary Figure S1. Conservation analysis of the variant identified in *TULP1* gene. **(A)** Multiple sequence alignment showing the conservation of amino acid Lys274 in TULP1 across evolution. **(B)** Comparative analysis of multiple sequence alignments illustrates the evolutionary conservation of the amino acid Lys459 in TULP1. These alignments were performed using HomoloGene (https://www.ncbi.nlm.nih.gov/homologene).

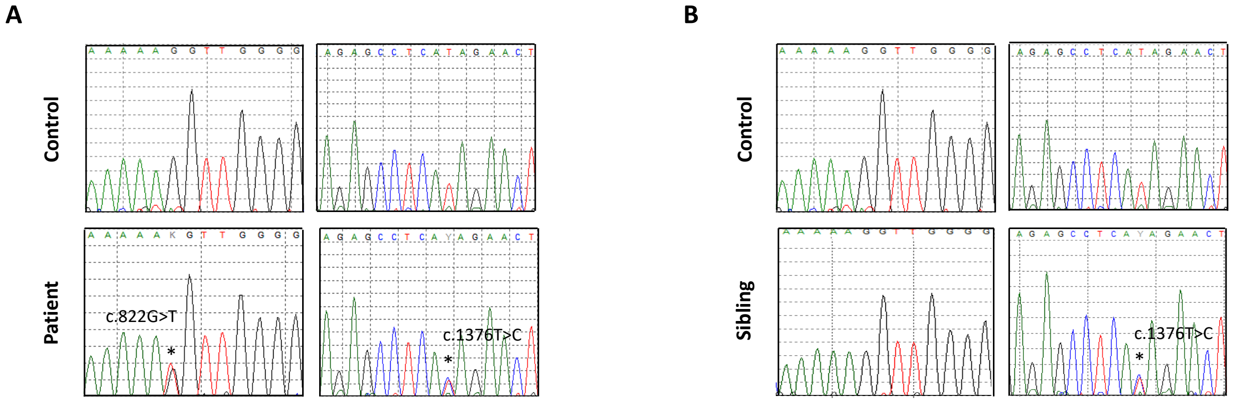

Supplementary Figure S2. Sanger sequencing performed to validate the identified variants within the *TULP1* gene and assess their segregation. **(A)** Sequencing results for exon 8 and 14 sequencing of the *TULP1* gene, confirming the presence of the c.822G>T and c.1376T>C variants. **(B)** Segregation analysis revealed that the patient's sibling is a carrier of the *TULP1*:c.1376T>C variant, thereby confirming that the identified variants in the patient are in a *trans* configuration.

3 Data availability

The datasets presented in this study can be found in the ENA online repository, accession number PRJEB70601.
